# Evaluation of Saliva Stability for NMR Metabolomics: Collection and Handling Protocols

**DOI:** 10.3390/metabo10120515

**Published:** 2020-12-19

**Authors:** Daniela Duarte, Beatriz Castro, Joana Leonor Pereira, Joana Faria Marques, Ana Luísa Costa, Ana M. Gil

**Affiliations:** 1 CICECO—Department of Chemistry, Aveiro Institute of Materials, Campus Universitário de Santiago, University of Aveiro, 3810-193 Aveiro, Portugal; danieladuarte@ua.pt (D.D.); bea.castro98@ua.pt (B.C.); 2 Dentistry Department, Faculty of Medicine, Institute of Paediatric and Preventive Dentistry, University of Coimbra, 3000-075 Coimbra, Portugal; joana.leonor.pereira@gmail.com (J.L.P.); aluisacosta@sapo.pt (A.L.C.); 3 GIBBO-Oral Biology and Biochemistry Research Group, CEMBDE-COCHRANE Portugal—Faculty of Dental Medicine, Universidade de Lisboa, 1649-003 Lisboa, Portugal; joanafariamarques@gmail.com

**Keywords:** NMR metabolomics, standard operating procedures, saliva, stability, storage

## Abstract

Maintaining a salivary metabolic profile upon sample collection and preparation is determinant in metabolomics. Nuclear magnetic resonance (NMR) spectroscopy was used to identify metabolite changes during short-term storage, at room temperature (RT)/4 °C/−20 °C, and after sample preparation, at RT/4 °C (mimicking typical clinical/laboratory settings). Interestingly, significant metabolic inter-individual and inter-day variability were noted, probably determining sample stability to some extent. After collection, no changes were noted at −20 °C (at least for 4 weeks). RT storage induced decreases in methylated macromolecules (6 h); lactate (8 h); alanine (12 h); galactose, hypoxanthine, pyruvate (24 h); sarcosine, betaine, choline, *N*-acetyl-glycoproteins (48 h), while acetate increased (48 h). Less, but different, changes were observed at 4 °C, suggesting different oral and microbial status at different temperatures (with a possible contribution from inter-individual and inter-day variability), and identifying galactose, hypoxanthine, and possibly, choline esters, as potential general stability indicators. After preparation, addition of NaN_3_ did not impact significantly on saliva stabilization, neither at RT nor at 4 °C, although its absence was accompanied by slight increases in fucose (6.5 h) and proline (8 h) at RT, and in xylose (24 h) at 4 °C. The putative metabolic origins of the above variations are discussed, with basis on the salivary microbiome. In summary, after collection, saliva can be stored at RT/4 °C for up to 6 h and at −20 °C for at least 4 weeks. Upon preparation for NMR analysis, samples are highly stable at 25 °C up to 8 h and at 4 °C up to 48 h, with NaN_3_ addition preventing possible early changes in fucose, proline (6–8 h), and xylose (24 h) levels.

## 1. Introduction

Metabolomics focuses on the high-throughput measurement of low-molecular-weight compounds comprised in a biological system [1,2]. The strategy has been proven as a valuable resource in medical research, having provided new mechanistic information on several diseases and proposed novel metabolic biomarkers, e.g., for type 1 diabetes *mellitus* (T1DM) [3], cardiovascular diseases [4], or cancer [5]. Applied to human disease, metabolomics usually involves the analysis of biofluids (mainly blood and urine, and more recently, saliva) through nuclear magnetic resonance (NMR spectroscopy) or hyphenated methods with mass spectrometry (MS) detection, two complementary analytical approaches, which provide a large amount of data subsequently handled by multivariate analysis (MVA) methodologies [6], to identify metabolic patterns consistent with the presence of disease.

Saliva metabolomics has been relatively underexplored, compared to blood or urine, although growing interest has been noted in the last decade [7,8], not only to find biomarkers of oral pathologies [9,10,11,12], but also of several other diseases, such as T1DM [13] and dementia [14,15]. Easy and non-invasive saliva collection adds to its interest in disease research, as it also enhances the compliance of elderly and children. It is important, therefore, to establish specific standard operating procedures (SOPs) to ensure saliva integrity throughout all steps of the metabolomics process. Indeed, this has been importantly carried out for urine and blood [16,17,18,19], and in relation to saliva, this need has been clearly recognized [8,20]. Several biochemical studies have investigated the impact of storage temperature on the stability of specific saliva specific components, namely steroids [21,22,23], immunoglobulins [23,24], peptides/proteins [25,26,27,28,29,30,31,32,33], and enzymes and metabolites, e.g., related to oxidative capacity [32,33]. Saliva stability at different temperatures has also been studied through metabolomic approaches, to our knowledge, only by MS-based strategies [34,35,36]. The effect of room temperature (RT), −20 °C and −80 °C on salivary metabolome, as viewed by liquid chromatography (LC) in tandem with MS, was assessed at times 0 and 1 month [34], the authors reporting that salivary metabolic profile seemed to be stable at all temperatures, up to 1 month. A subsequent targeted study considering possible biomarkers of oral squamous cell carcinoma [35] concluded that the levels of choline, betaine, pipecolinic acid, and L-carnitine remained stable at RT (after sample preparation) for up to 24 h, and during storage at −35 °C for 1 month. More recently, another targeted study aimed at evaluating the levels of salivary polyamines and amino acids, as possible cancer markers [36], during storage at RT (up to 4 h), in ice (up to 4 h), and at 22 °C, 4 °C, and −18 °C (up to 8 days). As expected, most detected polyamines and amino acids were observed to vary significantly in content during storage at RT (up to 4 h), whereas valine and *N*_1_-acetylspermidine were noted not to change significantly. Up to 8 days at RT, the changes in amino acids were generally larger than in polyamines, but ethanol addition was found to maintain sample integrity. No statistical variations were reported at the remaining temperatures under study [36]. Other protocol aspects have been addressed [7], this time by NMR metabolomics, and specifically concluding that up to four freeze–thaw cycles and different centrifugation conditions (750–15,000× *g* range) did not impact significantly on saliva composition, as viewed by NMR.

Here, we report the first untargeted ^1^H NMR metabolomics study, to our knowledge, of the effects of different storage temperatures and times on saliva composition. This study assesses salivary metabolic changes during storage in conditions, which are particularly relevant upon collection in a typical clinical setting (where −80 °C is not commonly available), and in the laboratory. In particular, we have considered saliva collected for several adult subjects, in the following conditions: (1) immediately after collection at 22 °C and 4 °C (up to 48 h, to mimic conditions in the clinic, where temporary storage out of a freezer is required) and at −20 °C (up to 4 weeks, for cases where a −80 °C freezer is not immediately available for ideal long-term storage); (2) after preparation for NMR analysis, with and without sodium azide (NaN_3_, a common bacteriostatic preservative), at 25 °C (up to 8 h, to mimic overnight NMR acquisition or standing time in non-refrigerated conditions prior to analysis), and at 4 °C (up to 48 h, to assess medium-term refrigerated storage prior or between analyses).

## 2. Results

**Figure 1:**
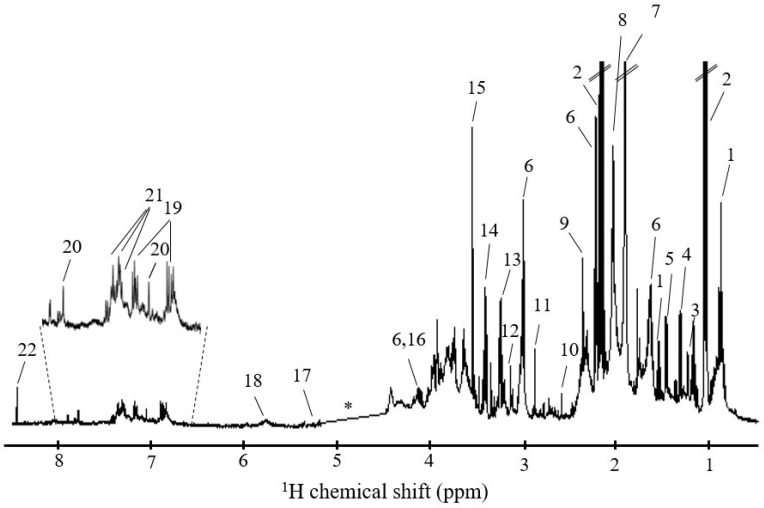
Typical 500 MHz ^1^H NMR spectrum of the saliva of a healthy (female) adult, with peak numbering identifying some assignments: 1, butyrate; 2, propionate; 3, fucose; 4, lactate; 5, alanine; 6, 5-aminopentanoate; 7, acetate; 8, *N*-acetyl (glycoproteins); 9, pyruvate; 10, methylamine; 11, trimethylamine; 12 dimethyl sulfone; 13, betaine; 14, taurine; 15, glycine; 16, proline; 17, xylose; 18, urea; 19, tyrosine; 20, histidine; 21, phenylalanine; 22, formate. * spectral region of water exclusion.

Figure 1 shows a typical ^1^H NMR spectrum of the saliva of a healthy (female) adult. The spectrum exhibits a large number of narrow peaks, arising from low molecular weight (M_w_) compounds (e.g., many amino acids, short chain fatty acids (SCFA), organic acids, carbohydrates and others, Table S1), often superimposed on broader resonances, which arise from high M_w_ compounds (mainly glycoproteins, the main protein components of saliva [37]). Based on the literature [12,38,39,40,41] and analysis of bidimensional NMR spectra of saliva, a total of *ca.* 50 metabolites could be identified (Table S1), although all of these have already been reported at least once in previous NMR reports (superscripts in Table S1) [12,38,39,40,41].

### 2.1. Saliva Stability after Collection, at 22 °C, 4 °C and −20 °C

**Figure 2:**
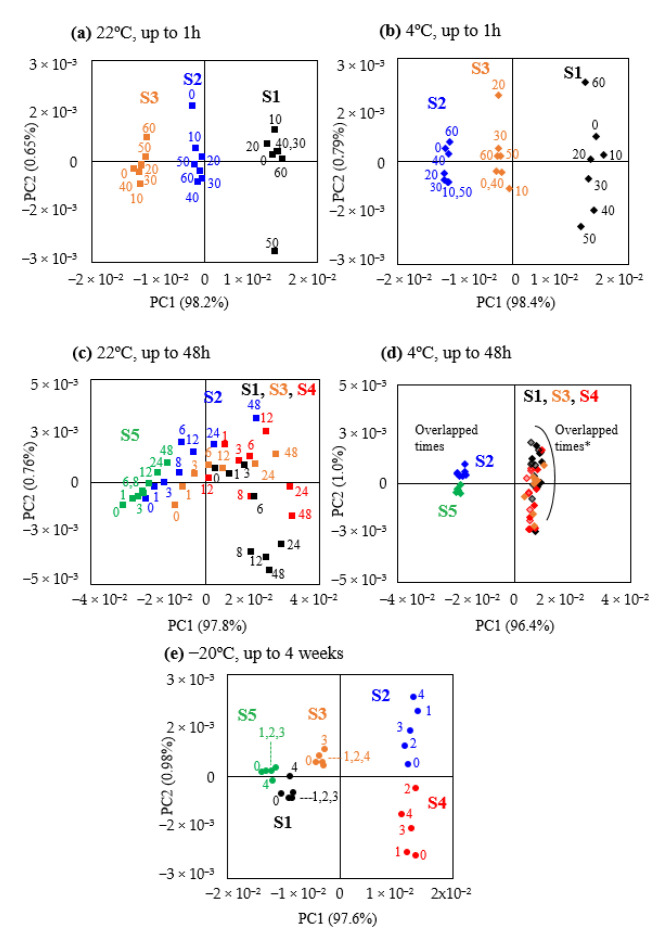
PCA scores plots for storage stability up to 1 h at (**a**) 22 °C and (**b**) 4 °C (numbers indicate minutes); up to 48 h at (**c**) 22 °C and (**d**) 4 °C (numbers indicate hours), *: group includes collections in different days for subjects 1 and 4 (darker/lighter symbols); (**e**) up to 4 weeks at −20 °C (numbers indicate weeks). Different individuals are represented by different colors: subject S1—black, subject S2—blue, subject S3—orange, subject S4—red, subject S5—green. Symbol shapes represent storage temperatures: 22 °C, ■; 4 °C, ♦; −20 °C, ●.

One of the aims of this study was to evaluate the stability of saliva samples temporarily stored at 22 °C, 4 °C, or −20 °C, before they can be transferred to the ideal conditions of long-term storage at −80 °C. Regarding short-term (up to 1 h) storage of saliva at 22 °C and 4 °C, PCA scores plots (Figure 2a,b) showed firstly that PC1 clearly separates the three individuals in terms of their saliva metabolic profile, at either temperature. Interestingly, as saliva was collected at different days for each of the experiments (22 °C and 4 °C), Figure 2a,b also illustrates that salivary metabolic profile depends on the day of collection (note the different relative positions of individuals S2 and S3), as some individuals donated saliva in different days for distinct parts of the study (see Section 4.1 of Materials and Methods). Comparison of NMR spectra for t = 0 showed that, generally, individuals positioned in positive PC1 in the score plots in Figure 2a,b,d exhibited decreased salivary levels of *N-*acetyl-glycoproteins (NAG), urea, and dimethyl sulfone and increased levels of trimethylamine, 5-aminopentanoate, acetate, propionate, leucine, and valine (interestingly, these samples also seem to exhibit larger intra-group dispersion, particularly at 4 °C, Figure 2b,d). Intra-group dispersion showed no significant differences, up to 1 h, between 22 °C and 4 °C results (with the exception of a larger dispersion at 4 °C for individual S1 in positive PC1, possibly due to sample collection on a different day). With basis on peak integration, no statistically relevant changes were noted, at either temperature, up to 1 h, hence establishing a high short-term (1 h) stability for human saliva, as viewed by NMR, at both 22 °C and 4 °C. When delays longer than 1 h (and up to 48 h) were considered prior to −80 °C storage (Figure 2c,d), 22 °C induced a clear gradual degradation of all samples, with some degree of inter-individual distinction still being noted, particularly in negative PC1 (subjects S2 and S5 in Figure 2c). Refrigeration improved saliva stability for all individuals, particularly for subjects S2 and S5 (Figure 2d). This shows that, for delays in the 1–48 h range, saliva stability seems to be individual-dependent and that, as expected, higher variability is shown for 22 °C, compared to 4 °C. Upon spectral visual inspection and peak integration, the metabolites with statistically relevant level variations could be identified (Table 1). At 22 °C, the first change noted was a decrease in a broad methyl resonance at 0.75 ppm (methylated large molecules, possibly proteins or other large M_w_ moieties), which became more significant as time evolved towards 48 h (Table 1). Lactate decreased from 8 h onwards, accompanied by decreasing levels of alanine at 12 h. At 24 h, additional changes comprised decreased levels of pyruvate, galactose, and hypoxanthine. At 48 h, acetate was observed to increase, accompanied by decreases in three amino acids (alanine, betaine, sarcosine), lactate and pyruvate, galactose, choline, NAG, and two unassigned resonances (Table 1). Within the above, decreased alanine, lactate and the broad methyl resonance at 0.75 ppm showed the strongest correlation with delay time at 22 °C (Table 1), consistently with their early statistically relevant decreases compared to original saliva (see boxplots in Figure S1). Under refrigerated conditions, as expected, saliva showed less changes over time, although an increase in xylose occurred as early as 6 h (Table 1 and Figure S2). After that, further changes are only noted at 24 h (increased tyrosine), followed by changes in eight metabolites at 48 h, notably defining a different profile, compared to 22 °C. Interestingly, only three varying metabolites were found in common between the two temperature conditions: galactose and hypoxanthine changing in opposite directions, whereas the resonance at 0.75 ppm (methylated large molecules) decreased under both conditions. This suggests that their levels may putatively be exploited, in the future, as individual-independent indicators of saliva integrity. It is possible that the remaining variations may reflect distinct biochemical processes occurring at the different temperatures, although a contribution from inter-individual variability in salivary profile may not be entirely ruled out.

To evaluate the suitability of a common freezer (at −20 °C) for temporary saliva storage, the profile of samples was investigated up to 48 h (data not shown), having indicated no relevant changes under such conditions. Considering a period of up to 4 weeks, PCA confirmed the large inter-individual difference in salivary profile (Figure 2e), with higher stability noted for three of the donors (S1, S3, S5), compared to the remaining ones. However, even though intra-group dispersion showed some individual-dependence, no metabolites were observed to vary with statistical relevance, indicating that storage at −20 °C, for up to 4 weeks, is generally acceptable for saliva, as viewed by NMR.

### 2.2. Stability of Saliva after Preparation for NMR Analysis

**Figure 3:**
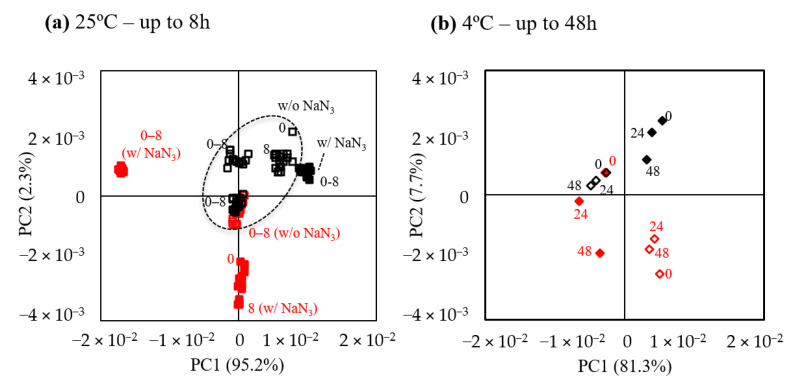
PCA scores plots for evaluation of saliva stability, after storage (−80 °C, 1–2 weeks), thawing and sample preparation at (**a**) 25 °C, with (■) and without NaN_3_ (☐); subject S1 (black) provided saliva in 3 different days for no azide assays and in 1 day for with azide assay; subject S4 (red) provided saliva in 1 day for no azide assays and in 2 different days for with azide assays; the ellipse indicates that all black symbols within the shape are open squares, thus corresponding to samples without azide. (**b**) 4 °C, with (◆) and without NaN_3_ (◇). Numbers represent hours. To evaluate both the effects of sodium azide and temperature, only subjects S1 (black) and S4 (red) were considered.

In order to evaluate the stability of saliva samples after preparation for NMR analysis, the effect of NaN_3_ on salivary metabolic profile was evaluated at 25 °C and 4 °C, over a period of 8 and 48 h, respectively (the choice of different times spans serves different aims, see Introduction and Materials and Methods section). Figure 3a shows that adding NaN_3_ does not significantly change intra-group dispersion at 25 °C for up to 8 h, conditions that mimic possible standing times prior to acquisition or overnight. Notably, stability seems high already in the absence of NaN_3_, although intra-group dispersion may be dependent on the individual and day of collection (see subject S4, in red, for whom three samples were collected in different days). At 4 °C, but for longer times (up to 48 h) (Figure 3b), intra-group spread appears independent of the presence of NaN_3_. In spite of the above results, peak integration for all samples without NaN_3_ revealed that slight changes may occur, on average, in saliva kept at 25 °C (Figure S3a), namely increases in fucose (significant from 6.5 h) and proline (significant from 8 h). At 4 °C, only an increase in xylose was observed at 24 h and, further, at 48 h (Figure S3b).

## 3. Discussion

### 3.1. Inter-Individual Variability in Salivary Metabolic Profile

**Figure 4:**
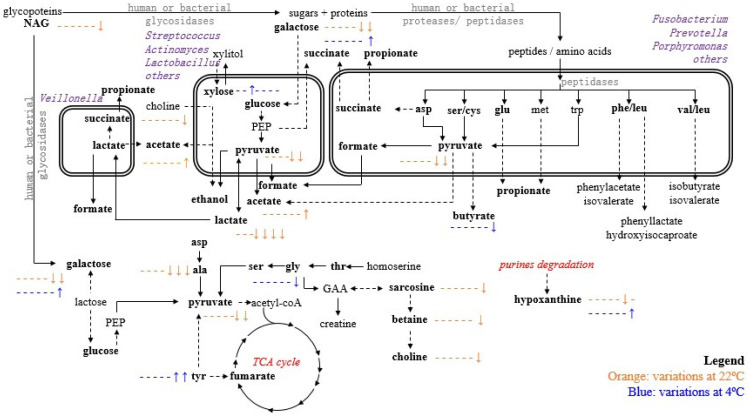
Main metabolic pathways affected by 4 °C and 22 °C storage after 1, 3, 6, 8, 12, 24, and 48 h. Metabolites in bold are those detected in this work, by NMR; blue, variations at 4 °C; orange, variations at 22 °C; the symbol - represents a time point where no variation was noted compared to 0 h; ↑ and ↓ represent metabolite level increased and decreased compared to 0 h, respectively; so that for instance the indication - - - - - ↑↑ expresses no changes at 1, 3, 6, 8, and 12 h, and increases at 24 and 48 h. Three-letter code used for amino acids; GAA, guanidinoacetate; NAG, *N*-acetyl of glycoproteins; PEP, phosphonenolpyruvate.

This study illustrated the significant extension of inter-individual variability in saliva metabolic profile, for healthy donors, as expected, considering the known large diversity of salivary microbiome [42]. One limitation of this work was that no oral or microbial examination could be carried out for the donors, so that specific correlations between metabolites and microbial diversity/load cannot be advanced at this stage. Figure 4 attempts to putatively correlate metabolic pathways with salivary microbiome, known to relate to individual oral and general health status [43,44,45] and depend on circadian rhythm or diets [46]. In the conditions of this work (healthy subjects and sampling at the same time of day), not only was salivary profile largely individual-dependent, but it also differed considerably between collection days. Sample pooling would circumvent this issue, although this was intentionally not carried out, in order to precisely evaluate inter-individual metabolic variation and possible correlation to saliva stability properties. Inter-individual differences involved mainly changes in the levels of NAG, urea, and dimethyl sulfone and increased levels of trimethylamine, 5-aminopentanoate, acetate, propionate, leucine, and valine. These differences could reflect individual-specific dietary or oral bacterial flora differences at baseline, since dimethyl sulfone is normally associated to methionine catabolism by bacteria from dietary products [47], while trimethylamine, acetate, 5-aminopentanoate, leucine, and valine could derive from bacterial metabolism of host or dietary choline [48] and salivary proteins, respectively [49,50]. The interesting observation that individuals with originally lower levels of NAG, urea, and dimethyl sulfone and higher levels of trimethylamine, 5-aminopentanoate, acetate, propionate, leucine, and valine seem to exhibit higher saliva variability, leads us to propose that such a profile may serve as a potential predictor of saliva metabolic stability (if confirmed in larger cohorts and eventually explained by additional microbial load assays).

### 3.2. Saliva Stability Post-Collection

In a clinical setting, it is often seldom that a −80 °C freezer is available for ideal immediate sample storage, and a short period of standing time at room temperature or under refrigeration may be required. Notably, saliva did not show statistically relevant changes at either 22 or 4 °C, up to 1 h. However, at 22 °C qualitative changes occur at 3 h, although statistically relevant changes only take place at 6 h, with a decrease in a methyl broad resonance. NMR peak linewidths inversely reflect molecular mobility; hence, we propose that such methyls arise from relatively larger molecules. As the typical NAG methyl resonances at 2.06 ppm only changes (decreases) at 48 h, we suggest that such molecules are not glycoproteins, but possibly other proteins or large molecules. This change may relate to the reported changes in total esterase (TEA) at 6 h at RT [32], thus putatively reflecting a decrease in esterified aliphatic moieties, such as acetylcholine [51,52] or larger choline esters.

Overall, the larger number of variations at 22 °C appear to express the role of bacterial proteases and saccharolytic pathways, explaining the decrease in pyruvate, galactose, alanine, and choline and the increase in acetate (Figure 4). The decrease in choline could be attributed to bacterial degradation of dietary choline by oral bacteria such as *Streptococcus sanguinis*, with the release of acetate and ethanol [48,53], possibly explaining the increase in acetate levels observed here (Figure 4). The reduced levels of pyruvate, galactose and lactate, reported at 22 °C at 8–48 h (lactate) and 24–48 h (galactose and pyruvate), may relate to involvement in the glycolytic pathway and their metabolization by oral bacteria: lactate can be metabolized to acetate by *Veillonella* [54], whereas lactate and butyric acid are expected to serve as substrates for lactate dehydrogenase produced by *Streptococcus, Lactobacillus*, and *Actinomyces* spp. [55]. The decreases in alanine, betaine, and sarcosine at 22 °C could be potentially attributed to their consumption by viable reminiscent bacteria as a nitrogen source [56].

The fact that saliva shows delayed degradation post-collection at 4 °C is expected, although a different profile of changes is noted (Figure 4). The most significant changes occur at 48h and comprise reversed variations in amino acids, the SCFA butyrate, galactose, and hypoxanthine, compared to 22 °C. It may be speculated that glycosylation of glycoproteins may occur at 4 °C, thus explaining the increase in xylose and galactose, which may remain elevated due to inhibition of saccharolytic bacteria at the lower temperature. In addition, changes in purine degradation pathways may take place to explain the reversed variations in hypoxanthine [57,58,59]. Indeed, the decrease noted in hypoxanthine at 22 °C could be attributed to the maintained activity of xanthine oxidase enzyme, which converts hypoxanthine into xanthine and uric acid [56]. At 4 °C, the activity of this enzyme may be inhibited, thus preventing hypoxanthine degradation into its oxidized forms, although purine degradation pathways are still expected to be active at 4 °C (for instance, promoting degradation of adenosine monophosphate (AMP) from endogenous or dietary sources) [57,58,59]. The significant increases in glycine and tyrosine at 4 °C could be attributed to endogenous proteolytic activity, as bacterial proteolytic degradation would not be expected at such low temperatures. Previous studies have reported proteolytic degradation after storage periods over 1 h, even at low temperatures, having suggested that storage at 4 °C (or lower) does not protect protease-sensitive biomarkers indefinitely, even when a protease inhibitor cocktail is used [25].

### 3.3. Saliva Stability Post-Preparation for NMR Analysis

Saliva is here observed to be remarkably stable after preparation, at least up to 8 h at 25 °C, so that adding NaN_3_ does not seem to be necessary (although inter-day variability may contribute somewhat to determining microbial stability). Still, slight changes were noted in fucose and proline, probably arising from proteolytic cleavage of endogenous glycoproteins, such as glycosylated proline-rich proteins or mucins. Under refrigeration for up to 2 days, saliva also exhibits high stability, again seemingly not requiring NaN_3_ addition and only exhibiting an increase in xylose (after 24 h), probably also due to glycoproteins hydrolysis. In addition, it is worth recalling that some Gram-positive bacteria, namely *Streptococci* and *Lactobacilli*, have been reported to be intrinsically resistant to the oxidant activity of sodium azide [60]. Since these genera are dominant in cariogenic and saccharolytic dental biofilms, it is plausible that they may be metabolically viable in samples with a higher bacterial load. In this respect, the additional evaluation of the relative bacterial load of the donors would contribute importantly to validate such hypothesis.

## 4. Materials and Methods

### 4.1. Sample Collection

**Figure 5:**
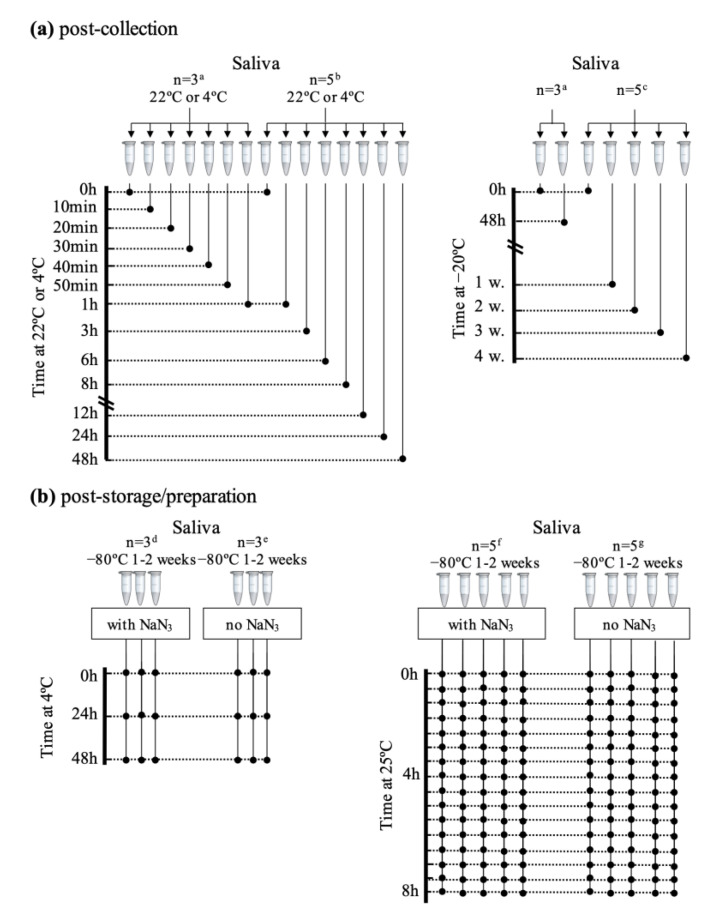
Schematic workflow for the studies of saliva compositional stability (**a**) immediately after collection, at 22 °C (0 to 48 h), 4 °C (0 to 48 h), and −20 °C (0 to 4 weeks), ^a^ donors: subjects S1–S3, ^b,c^ donors: subjects S1–S5; (**b**) after storage (−80 °C, 1–2 weeks), thawing and preparation for NMR analysis, with and without sodium azide, at 4 °C (^d^ donors: subjects S1, S2, and S4, ^e^ donors: subjects S1, S3, and S4), and at 25 °C; (^f^ donors: subjects S1, S4, and S5, ^g^ donors: subjects S1, S3, and S4). Please note that some of the subjects donated saliva more than once, in different days, and that no samples were pooled, in order for inter-individual and inter-day variability to be assessed.

All saliva samples were collected under the approval of the Ethical Committee of the Hospital Center of Coimbra (CHUC-091-17, dated 25 June 2018) and signed informed consent forms were obtained from each participating subject. Saliva was collected from 5 healthy female donors (S1 to S5), aged between 21 and 29 years old and body mass indexes in the normal 18.5–21 kg/m^2^ range. Some of these subjects provided saliva samples in different days, for different parts of the study (Figure 5). Unstimulated (or resting) saliva samples (i.e., saliva collected in the absence of any mechanical or other stimuli) were collected through passive drool, by expectoration for up to 10 min, or until *ca.* 6 mL of saliva was obtained, according to previous recommendations [12,61]. In order to minimize interference of different stages of the circadian rhythm, all collections were carried out in the midmorning period (*ca*. 11:00 a.m.), and donors were requested to abstain from eating, drinking, smoking, or tooth brushing in the last 1.5 h prior to collection. All collected samples were gently stirred to ensure homogeneity and split into the required number of aliquots. Samples were intentionally not pooled, in order to assess inter-individual variability either in basal salivary profile and/or its stability. For short- (1 h), medium-term (48 h), and post-collection studies (Figure 5a, left), all donors from S1 to S5 provided a sample, which was split into the necessary number of aliquots. For each donor, one of the aliquots was stored at −80 °C immediately (t = 0 h), and the remaining aliquots were stored during different time periods at either 22 °C (22 ± 2 °C) or 4 °C (before subsequent immediate −80 °C storage). The short- and medium-term assays included NMR acquisition at shorter periods (0,10, 20, 30, 40, 50, 60 min) and at longer periods (0, 1, 3, 6, 8, 12, 24, 48 h), respectively (before −80 °C storage). For evaluating stability at −20 °C (Figure 5a, right), one of the aliquots was immediately stored at −80 °C (t = 0), and the remaining were stored at 48 h and 1, 2, 3, and 4 weeks (prior to −80 °C storage). To assess the degree of sample degradation after immediate storage at −80 °C (1–2 weeks) on thawing and preparation for NMR acquisition (Figure 5b), two sample sets (with and without the NaN_3_) were prepared to assess effects of storage at 4 °C (at t = 0, 24 h and 48 h) and at 25 °C up to 8 h (with 16 spectra acquired consecutively up to 8 h, approximately every 30 min). In this part of the work, some of the subjects donated saliva in different days, for different assays, hence the discussion of the effects of sodium azide for instance being mainly centered in subjects 1 and 4, for which complete sets of samples were acquired to assess sodium azide and temperature effects.

### 4.2. Sample Preparation for NMR Analysis

After thawing, each sample was centrifuged (1 h, 4 °C, 10,000 rpm or 9184× *g*, in a Sigma 2-16P centrifuge with r = 82 mm). Then, to 400 μL of the supernatant, 300 μL of sodium phosphate buffer were added: 70 mM Na_2_HPO_4_/NaH_2_PO_4_, containing 0.1% (*m*/*v*) Na^+^/3-trimethylsilyl-propionate (TSP) and 2 mM NaN_3_, except in the case of samples aimed at studying the effect of sodium azide absence (Figure 5b). After vortexing, 600 μL of the mixture were transferred to 5 mm NMR tubes.

### 4.3. NMR Spectroscopy

For each saliva sample, a ^1^H NMR spectrum was recorded on a Bruker Avance III spectrometer, operating at 500.13 MHz for proton, at 298 K, using a *noesypr1d* pulse sequence (Bruker library) with an 8.10–8.97 µs 90 degree pulse, 100 ms mixing time, water suppression (with a power level of 45.57 dB and offset frequency of ca. 2350 Hz) during mixing time and relaxation delay (RD 4 s). The 90-degree pulse length and water suppression conditions were optimized for each sample. A total of 256 transients were acquired into 64 k data points, with spectral width of 10,000 Hz, and 3.28 s acquisition time. Each free induction decay (FID) was multiplied by a 0.3 Hz exponential function prior to Fourier transformation. Spectra were manually phased, and baseline-corrected and chemical shifts were referenced internally to TSP (δ 0.0). Peak assignments were carried out based on literature reports [12,38,39,40,41], Human Metabolome Database [62], 2D NMR experiments (total correlation spectroscopy, TOCSY, and heteronuclear single quantum correlation spectroscopy, HSQC on selected samples, following acquisition conditions described elsewhere [12]), and consultation of the Bruker Biorefcode database.

### 4.4. Statistical Analysis

After excluding the water region (4.50–5.19 ppm), the spectra were aligned by recursive segment-wise peak alignment [63] and normalized to total area, accounting for sample concentration differences. Principal component analysis (PCA) was performed after testing different types of scaling (centered, unit variance and Pareto) and having selected the former (SIMCA-P 11.5, Umetrics, Sweden). All relevant peaks were integrated (Amix 3.9.14, Bruker BioSpin, Rheinstetten, Germany), normalized to spectral total area, and variations were assessed through effect size (ES), adjusted for low sample sizes [64] and *p*-values (Wilcoxon test). Metabolite variations were considered significant if |ES| > ES_error_ and *p*-value < 0.05. Spearman correlation analysis was also performed to correlate each metabolite with time length storage for each condition under study. All statistical tests and boxplots were performed using the R-statistical software and MATLAB (8.3.0, MathWorks).

## 5. Conclusions

With this work, we aimed at mimicking realistic conditions regarding saliva sampling in the clinic (post-collection) and in the laboratory (post-preparation, particularly for NMR), in order to propose best practices regarding saliva storage in the short and medium term. While noting a significant inter-individual and inter-day variability in the metabolic profile of saliva post-collection, our results showed that, after collection, saliva may be kept at 22 °C or 4 °C for up to 6 h, after which some metabolite levels start to change (in greater number at 22 °C, as expected). For longer periods, saliva is best stored at −20 °C for at least 4 weeks (alternatively to −80 °C). We propose that the metabolite changes noted should reflect a distinct interplay of oral and microbial metabolisms at the different temperatures, in tandem with a possible contribution from inter-individual variability. Only galactose, hypoxanthine, and possibly, methylated choline esters were found varying at both 22 °C and 4 °C, suggesting their levels as potential individual-independent indicators of saliva integrity. The stability of saliva after preparation for NMR analysis was evaluated at 25 °C (for 8 h, to mimic overnight acquisition or standing time, without refrigeration) and at 4 °C (for 48 h, to mimic refrigerated standing time before acquisition). In both conditions, saliva stability was high and, although addition of sodium azide to maintain sample microbiological integrity was found not to have a significant effect on saliva stability, its absence may induce early (6–8 h) increases in fucose, xylose, and proline levels.

One limitation of this work relates to the low number of subjects, although multiple collections were made when possible (thus enabling inter-day variability to be assessed). A second important limitation relates to the absence of in tandem oral and microbiological examinations of the donors. Although all subjects regularly attended routine dental appointments, their examination by a calibrated trained dentist would have evaluated important oral status indicators (biofilm accumulation, dental status, and oral soft tissue alterations), which would aid in the interpretation of the considerable inter-individual variability found in saliva profile and stability. Nevertheless, we believe that our results help to establish useful guidelines as to how to suitably store saliva samples in a clinical setting immediately post-collection (at RT/4 °C for up to 6h and at −20 °C, for at least 4 weeks) or after preparation in the lab (at 25 °C up to 8 h and at 4 °C up to 48 h, with recommended use of sodium azide), thus ensuring sample integrity for NMR metabolomics.

## Figures and Tables

**Table 1:** Metabolites varying significantly in human saliva (female donors), during storage at 22 °C and 4 °C, up to 48 h. Only metabolites with |ES| > ES_error_ and *p*-values < 0.05 were considered. ^a^ overlapped resonances may contribute slightly to variations in this peak; ^b^ metabolites varying at both temperatures, although not necessarily in the same direction; NAG, *N*-acetyl of glycoproteins; SCFA, short chain fatty acids; U*i*, unassigned resonance *i* ordered by chemical shift; *r*^2^: correlation coefficient. Chemical shifts shown in the second column correspond to the peaks used for integration.

T (°C)	Metabolite (δ H/ppm, Multiplicity)	6 h vs. 0 h		8 h vs. 0 h		12 h vs. 0 h		24 h vs. 0 h			48 h vs. 0 h	Correlation	
		Effect Size	*p*-Value	Effect Size	*p*-Value	Effect Size	*p*-Value	Effect Size	*p*-Value	Effect Size	*p*-Value	*r* ^2^	*p*-Value
22 °C	Amino acids												
	alanine (1.48, d)	-	-	-	-	−1.62 ± 1.33	7.92 × 10^−3^	−1.90 ± 1.39	1.6 × 10^−2^	−1.82 ± 1.38	3.2 × 10^−2^	−0.66	4.2 × 10^−6^
	betaine (3.26, s)	-	-	-	-	-	-	-	-	−1.51 ± 1.30	3.3 × 10^−2^	−0.58	9.5 × 10^−5^
	sarcosine ^a^ (2.74, s)	-	-	-	-	-	-	-	-	−1.67 ± 1.34	7.9 × 10^−3^	−0.56	1.8 × 10^−4^
	SCFA												
	acetate (1.92, s)	-	-	-	-	-	-	-	-	1.57 ± 1.31	2.6 × 10^−2^	0.52	5.9 × 10^−4^
	Organic acids												
	lactate (4.11, q)	-	-	−1.33 ± 1.26	3.22 × 10^−2^	−1.75 ± 1.36	3.3 × 10^−2^	−2.07 ± 1.44	7.9 × 10^−3^	−2.53 ± 1.56	7.9 × 10^−3^	−0.64	8.1 × 10^−6^
	pyruvate ^a^ (2.38, s)	-	-	-	-	-	-	−1.41 ± 1.28	3.2 × 10^−2^	−1.70 ± 1.35	1.6 × 10^−2^	−0.58	8.7 × 10^−5^
	Carbohydrates												
	galactose (5.27, d)	-	-	-	-	-	-	−1.67 ± 1.34	3.2 × 10^−2^	−2.05 ± 1.44	7.9 × 10^−3^	−0.50	1.0 × 10^−3^
	Other compounds												
	choline (3.20, s)	-	-	-	-	-	-	-	-	−2.04 ± 1.43	3.2 × 10^−2^	−0.49	1.5 × 10^−3^
	hypoxanthine (8.19, s)	-	-	-	-	-	-	−1.44 ± 1.43	3.2 × 10^−2^	-		−0.47	2.2 × 10^−3^
	NAG ^a^ (2.06, s)	-	--	-	-	-		-	-	−1.85 ± 1.38	3.2 × 10^−2^	−0.55	2.0 × 10^−4^
	Unassigned resonances												
	U1 (0.75, br)	−1.62 ± 1.33	3.2 × 10^−2^	−1.73 ± 1.35	3.2 × 10^−2^	−1.41 ± 1.28	3.2 × 10^−2^	−2.50 ± 1.57	7.9 × 10^−3^	−2.80 ± 1.66	7.9 × 10^−3^	−0.66	3.5 × 10^−6^
	U3 (3.22, s)	-		-	-	-	-	-		−1.60 ± 1.32	1.6 × 10^−2^	−0.50	9.0 × 10^−4^
4 °C	Amino acids												
	glycine (3.56, s)	-	-	-	-	-	-	-	-	1.33 ± 1.14	3.5 × 10^−2^	0.46	5.1 × 10^−4^
	tyrosine (6.90, d)	-	-	-	-	-	-	1.79 ± 1.23	1.4 × 10^−2^	1.97 ± 1.27	4.7 × 10^−3^	0.57	7.3 × 10^−6^
	SCFA												
	butyrate (1.55, q)	-	-	-	-	-	-	-	-	−1.23 ± 1.12	4.8 × 10^−2^	−0.43	1.3 × 10^−3^
	Carbohydrates												
	galactose ^b^ (5.27, d)	-	-	-	-	-	-	-	-	1.99 ± 1.27	8.2 × 10^−3^	0.46	5.9 × 10^−4^
	xylose (5.21, d)	1.26 ± 1.16	4.1 × 10^−2^	-	-	-	-	-	-	1.48 ± 1.16	2.2 × 10^−2^	0.45	6.7 × 10^−4^
	Other compounds												
	hypoxanthine ^b^ (8.19, s)	-	-	-		-	-	-	-	1.13 ± 1.10	3.5 × 10^−2^	0.34	1.4 × 10^−2^
	Unassigned resonances												
	U1 ^b^ (0.75, br)	-	-	-	-	-	-	-	-	−2.18 ± 1.32	4.7 × 10^−3^	−0.57	8.3 × 10^−6^
	U2 (0.84, s)	-	-	-	-	-	-	-	-	−1.54 ± 1.17	2.2 × 10^−2^	−0.40	2.7 × 10^−3^

## Supplementary Materials

The following are available online at https://www.mdpi.com/2218-1989/10/12/515/s1. Figure S1: Boxplots showing the main metabolites varying in saliva (post-collection) at 22 °C, up to 48 h, Figure S2: Boxplots showing the main metabolites varying in saliva (post-collection) at 4 °C, up to 48 h, Figure S3: Boxplots of metabolite variations noted post-preparation, in the absence of sodium azide and at 25 °C, Table S1: bearing the list of peak assignments in the 1H NMR spectrum of saliva of a healthy adult female subject.
