# Supplementary material for: Evaluation of Saliva Stability for NMR Metabolomics: Collection and Handling Protocols
Source: Metabolites. 2020 Dec 19;10(12):515. doi: 10.3390/metabo10120515 (PMC7766053; doi:10.3390/metabo10120515)
Supplement: Supplementary file 1 [file metabolites-10-00515-s001.pdf]

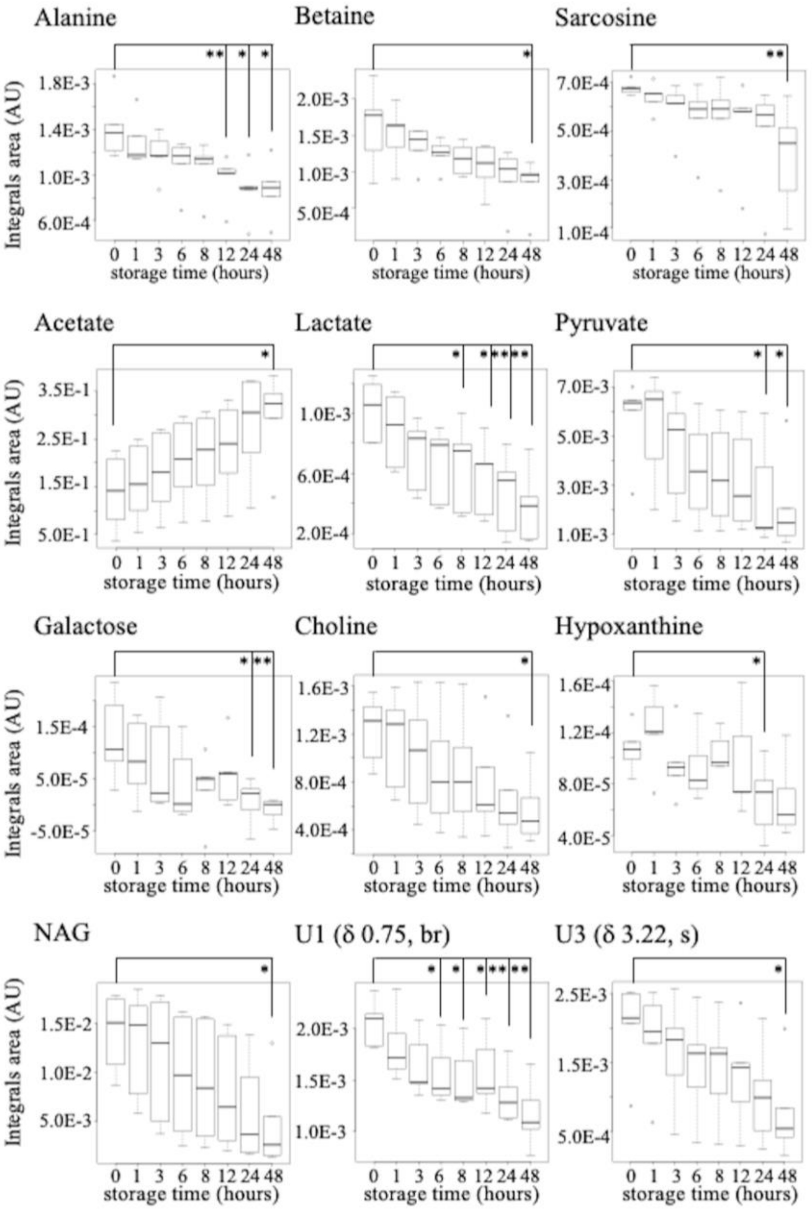


**Figure S1.** Boxplots showing the main metabolites varying in saliva (post-collection) at 22ºC, up to 48h.

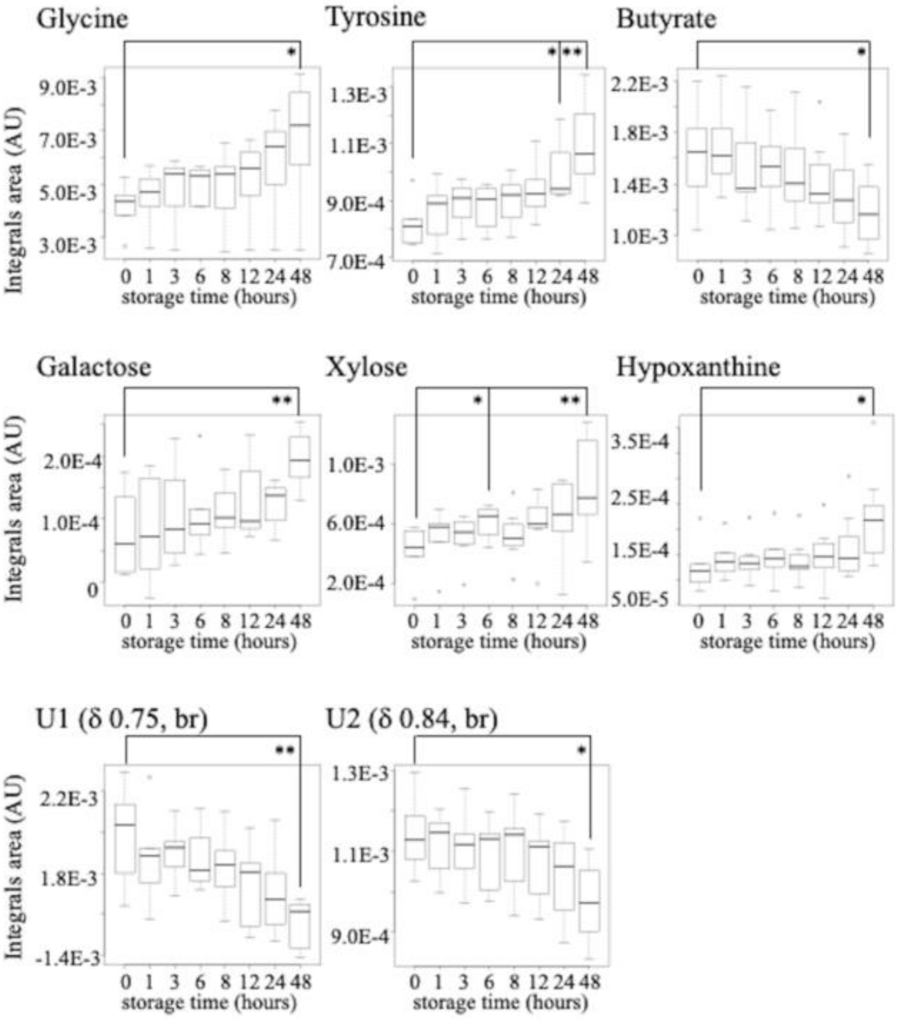


**Figure S2.** Boxplots showing the main metabolites varying in saliva (post-collection) at 4ºC, up to 48h.

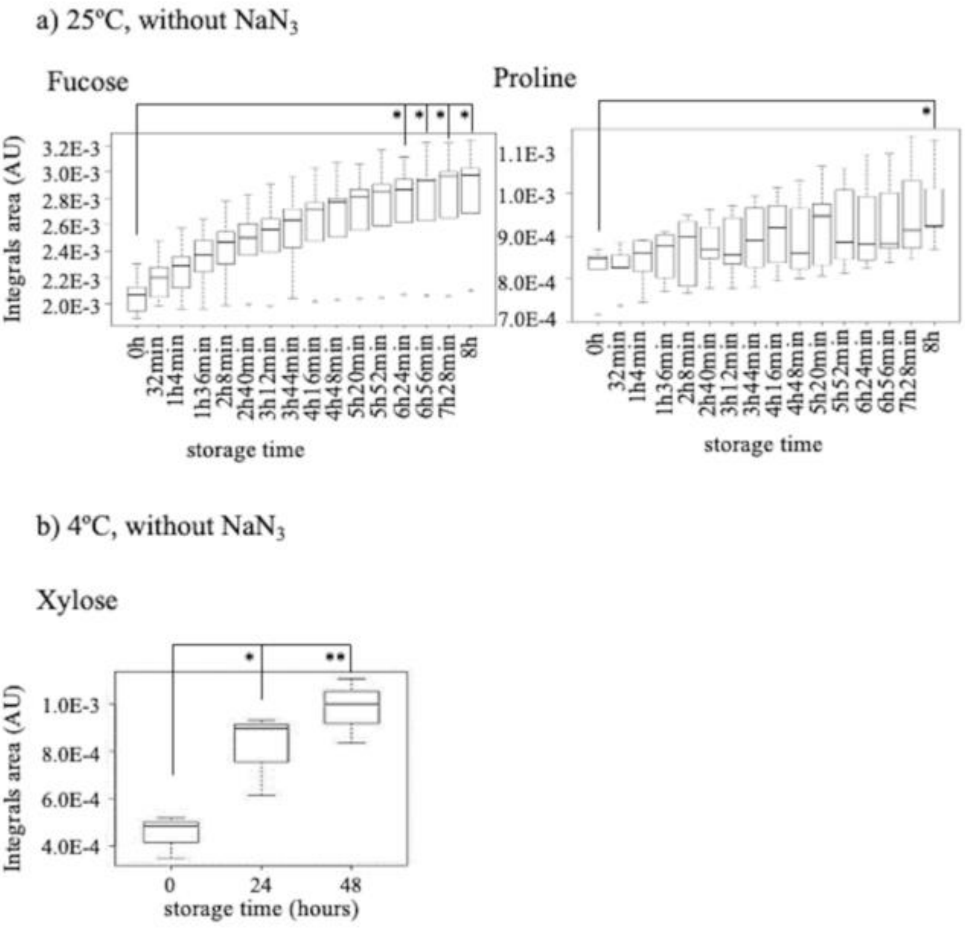


**Figure S3.** Boxplots of metabolite variations noted post-preparation, in the absence of sodium azide and at 25°C.

**Table S1.** Peak assignments in the $^{1}H$ NMR spectrum of saliva of healthy adult female subjects. s: singlet, d: doublet, t: triplet, q: quartet, dd: doublet of doublets, m: multiplet, br: broad signal. SCFA, short chain fatty acids; [a,b,c,d] compounds previously reported in NMR studies of unstimulated saliva of healthy adults in references [38] to [41], respectively; [e] compound detected by NMR in saliva of children [12]. U*i*: unassigned resonances *i*, for which significant variations were noted at least in one of the conditions considered. HMDB ID: compound ID number according to the Human Metabolome Database [62].

| Metabolite | HMDB ID | $\delta_{H\ ppm}$ (multiplicity) |
|---|---|---|
| *Amino acids* | | |
| alanine [a, b, c] | HMDB0000161 | 1.48 (d), 3.76 (q) |
| aspartate [a, c] | HMDB0000191 | 2.69 (dd), 2.83 (dd), 3.90 (dd) |
| glycine [a, b, c] | HMDB0000123 | 3.56 (s) |
| glutamate [a, c] | HMDB0003339 | 2.04 (m), 2.11 (m), 2.34 (m), 3.74 (dd) |
| glutamine [a, c] | HMDB0000641 | 2.12 (m), 2.44 (m), 3.76 (t) |
| histidine [a, b, c] | HMDB0000177 | 3.02 (m,), 3.15 (s), 7.05 (s), 7.75 (s) |
| isoleucine [a, c] | HMDB0000172 | 0.92 (t), 0.99 (d), 1.24 (m), 1.45 (m), 1.96 (m), 3.66 (d) |
| leucine [a, b, c] | HMDB0000687 | 0.94 (t), 1.7 (m), 3.72 (m) |
| lysine [a, c] | HMDB0000182 | 1.46 (m), 1.71 (m), 1.89 (m), 3.02 (t), 3.74 (t) |
| phenylalanine [a, b, c] | HMDB0000159 | 3.19 (m), 3.98 (dd), 7.32 (d), 7.36 (m), 7.42 (m) |
| proline [a, c] | HMDB0000162 | 1.99 (m), 2.06 (m), 2.35 (m), 3.33 (dt), 3.41 (dt), 4.16 (dd) |
| sarcosine [a, b, c] | HMDB0000271 | 2.74 (s), 3.60 (s) |
| taurine [a, b, c] | HMDB0000251 | 3.27 (t), 3.42 (t) |
| threonine [a, c] | HMDB0000167 | 1.32 (d), 3.57 (d), 4.26 (m) |

| | | |
|---|---|---|
| tyrosine [a, b, c] | HMDB0000158 | 3.06 (dd), 3.12 (dd), 3.95 (dd), 6.90 (d), 7.20 (d) |
| valine [a, c] | HMDB0000883 | 0.99 (d), 1.05 (d) |
| ***SCFA*** | | |
| acetate [a, b, c] | HMDB0000042 | 1.92 (s) |
| butyrate [a, c] | HMDB0000039 | 0.89 (t), 1.56 (m), 2.14 (t) |
| formate [a, b, c] | HMDB0000142 | 8.46 (s) |
| propionate [a, b, c] | HMDB0000237 | 1.06 (t), 2.18 (q) |
| ***Organic acids*** | | |
| 4-hydroxyphenyllactate [c] | HMDB0000755 | 2.87 (dd), 3.04 (dd), 4.35 (dd), 6.85 (d), 7.17 (d) |
| 5-aminopentanoate [c] | HMDB0003355 | 1.65 (.m), 2.23 (t), 3.00 (t) |
| ascorbate [a] | HMDB0000044 | 3.76 (m); 4.01 (m); 4.52 (d) |
| lactate [a, b, c] | HMDB0000190 | 1.32 (d), 4.11 (q) |
| pyruvate [a, b, c] | HMDB0000243 | 2.38 (s) |
| succinate [a, c] | HMDB0000254 | 2.41 (s) |
| ***Carbohydrates*** | | |
| $\alpha$-fucose [c] | | 1.21 (d), 1.25 (d), 3.45 (dd), 3.64 (dd), 3.79 (m), 4.56 (d) |
| $\alpha$-galactose [a, c] | HMDB0000143 | 3.48 (dd), 3.64 (dd), 3.72 (m), 3.82 (m), 3.92 (d), 3.96 (d), 4.07 (t), 5.27 (d) |
| $\alpha$-glucose [a, b, c] | HMDB0000122 | 3.23 (dd), 3.39 (m), 3.45 (m), 3.52 (dd), 3.72 (m), 3.82 (m), 3.88 (dd), 4.63 (d), 5.23 (d) |
| sucrose [b, c] | HMDB0000258 | 3.46 (t), 3.55 (dd), 3.67 (s), 3.75 (t), 3.82 (m), 3.87 (dd), 3.89 (dd), 4.04 (t), 4.21 (d), 5.42 (d) |
| xylose [e] | HMDB0000098 | 3.23 (dd), 3.33 (dd), 3.42 (t), 3.53 (dd), 3.63 (m), 3.93 (dd), 4.58 (d), 5.21 (d) |
| ***Amines*** | | |
| dimethylamine [a, c] | HMDB0000087 | 2.73 (s) |
| hypoxanthine [a, c] | HMDB0000157 | 8.19 (s), 8.20 (s) |
| methylamine [a, c] | HMDB0000164 | 2.61 (s) |
| trimethylamine [a, c] | HMDB0000906 | 2.89 (s) |
| ***Other compounds*** | | |
| acetone [a, b, c] | HMDB0001659 | 2.23 (s) |
| acetoin [d] | HMDB0003243 | 1.39 (d) |
| allantoin [a] | HMDB0000462 | 5.39 (s) |
| choline [a, c] | HMDB0000097 | 3.20 (s), 3.52 (m), 4.07 (m) |
| dimethyl sulfone [c] | HMDB0004983 | 3.15 (s) |
| ethanol [a, b, c] | HMDB0000108 | 1.19 (t), 3.65 (q) |
| methanol [a, b, c] | HMDB0001875 | 3.36 (s) |
| methylguanidine [a, c] | HMDB0001522 | 2.83 (s), 3.36 (s) |
| *N*-acetyl-glycoproteins [a] | | 2.06 (s) |
| propylene glycol [b,c] | HMDB0001881 | 1.15 (d), 3.43 (dd), 3.53 (dd), 3.87 (m) |
| putrescine | HMDB0001414 | 3.04 (m), 1.76 (m) |
| uracil [c] | HMDB0000300 | 5.79 (d), 7.52 (d) |
| urea [b,c] | HMDB0000294 | 5.78 (br) |
| ***Unassigned resonances*** | | |
| U1 | | 0.75 (br) |
| U2 | | 0.84 (s) |
| U3 | | 3.22 (s) |
